# Analysis of Thiodiphenol in Rat Urine as a Biomarker of Exposure to Temephos

**DOI:** 10.3390/jox14040100

**Published:** 2024-12-04

**Authors:** Miao-Ling Shih, Ma. de Lourdes López-González, Marisela Uribe-Ramírez, Aurora Elizabeth Rojas-García, Francisco Alberto Verdín-Betancourt, Adolfo Sierra-Santoyo

**Affiliations:** 1 Departamento de Toxicología, Centro de Investigación y de Estudios Avanzados del Instituto Politécnico Nacional (Cinvestav-IPN), Av. IPN 2508, Col. San Pedro Zacatenco, Ciudad de México 07360, Mexico; 2 Laboratorio de Contaminación y Toxicología Ambiental, Secretaría de Investigación y Posgrado, Universidad Autónoma de Nayarit, Tepic 63000, Nay., Mexico; 3 Unidad Especializada de Ciencias Ambientales, Centro Nayarita de Innovación y Transferencia de Tecnología, Av. Emilio M. González S/N, Ciudad del Conocimiento, Tepic 63173, Nay., Mexico

**Keywords:** temephos, thiodiphenol, BPS, biomarkers, organophosphorus pesticides

## Abstract

Temephos is an organophosphorus pesticide widely used as a larvicide in public health campaigns to control vector-borne diseases. Data on the urinary elimination of temephos metabolites are limited, and there is no validated biomarker of exposure for its evaluation. This study aimed to determine the urinary excretion kinetics of temephos and its metabolites in adult male rats. Hence, adult male Wistar rats were administered orally with a single dose of temephos (300 mg/kg). Urine samples were collected at different time intervals after dosing and enzymatically hydrolyzed using β-glucuronidase/sulfatase from *H. pomatia*. The metabolites were extracted and analyzed by HPLC-DAD. The metabolites detected were 4,4′-thiodiphenol (TDP), 4,4′-sulfinyldiphenol (SIDP), 4,4′-sulfonyldiphenol (SODP), or bisphenol S (BPS), a non-identified metabolite, and only traces of the parent compound. The mean urine concentrations of metabolites were used for kinetic analysis. Urinary levels of TDP were fitted to a two-compartmental model, and its half-lives (*t*_1/2 Elim-U_) were 27.8 and 272.1 h for the first and second phases, respectively. The *t*_1/2 Elim-U_ of BPS was 17.7 h. TDP, the main metabolite of temephos, was eliminated by urine and is specific and stable. Therefore, it may be used as a biomarker of temephos exposure.

## 1. Introduction

Temephos is an organophosphorothioate widely used as a larvicide in public health campaigns to control some vector-borne diseases, which are mainly transmitted by *Aedes aegypti*, such as dengue, Zika, and chikungunya. The World Health Organization (WHO) recommended its use in drinking water at concentrations not exceeding 1 mg/L; such concentrations do not affect its taste nor produce toxic reactions in humans and other mammals [1,2].

The everyday use of this safe larvicide in dengue-endemic sites implies frequent exposure in humans through the application by workers for the treatment of potable water. The limited information on its toxicokinetics and toxicity contributes to the extensive use of this pesticide, which has been used many times without control or overuse. Recently, Verdin-Betancourt et al. [3] reported the toxicokinetics of this pesticide in blood. The authors found that temephos is rapidly absorbed in rats after the administration of a single oral dose of 300 mg/kg; it has a high distribution volume, suggesting that the compound has a very high affinity with the extravascular tissues. The parent compound was distributed to the liver, kidneys, and brain and is mainly found in adipose tissue, where it has an extremely high affinity for accumulation compared to other organs. After repeated exposures, temephos was also detected in the placenta, testis, and epididymis [4,5].

**Figure 1:**
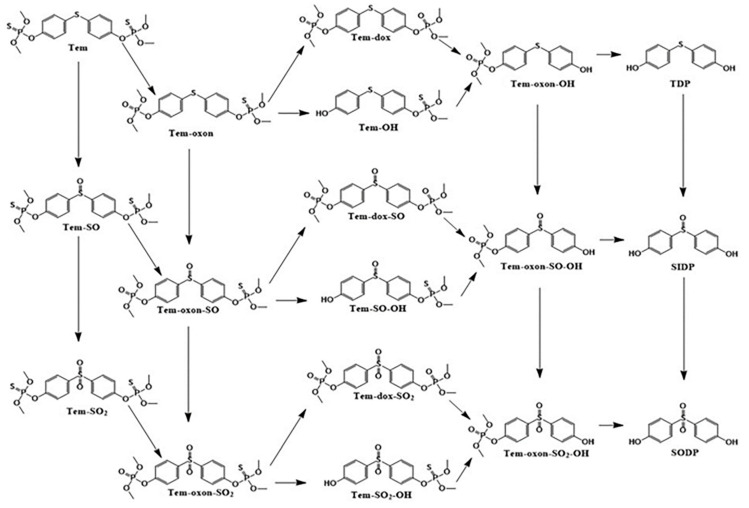
Proposed biotransformation pathway of temephos in the rat. Tem, temephos; Tem-SO, temephos sulfoxide; Tem-SO_2_, temephos sulfone; Tem-oxon, temephos oxon; Tem-oxon-SO, temephos oxon sulfoxide; Tem-oxon-SO_2_, temephos-oxon-sulfone; Tem-dox, temephos dioxon; Tem-OH, temephos monohydrolyzed; Tem-dox-SO, temephos dioxon sulfoxide; Tem-SO-OH, temephos sulfoxide monohydrolyzed; Tem-dox-SO_2_, temephos-dioxon-sulfone; Tem-SO_2_-OH, temephos sulfone monohydrolyzed; Tem-oxon-OH, temephos oxon monohydrolyzed; Tem-oxon-SO-OH, temephos oxon sulfoxide monohydrolyzed; Tem-oxon-SO_2_-OH, temephos oxon sulfone monohydrolyzed; TDP, 4,4′-thiodiphenol; SIDP, 4,4′-sulfinyldiphenol; and SODP, 4,4′-sulfonyldiphenol or bisphenol S (BPS) (modified from [3]).

Temephos is metabolized through oxidative desulfurization, *S*-oxidation, and hydrolysis reactions (dephosphorylation); it turns into at least eighteen metabolites in rats (Figure 1) [3], and a similar number of metabolites was described in humans in an in silico prediction study [6]. Some metabolites are conjugated with phase II enzymes and are eliminated in rat urine as sulfate and glucuronide conjugates, especially dephosphorylated metabolites, such as 4,4′-thiodiphenol (TDP), 4,4′-sulfinyldiphenol (SIDP), 4,4′-sulfonyldiphenol (SODP), thiodiphenol sulfone (TDP-SO_2_), or bisphenol S (BPS) [7,8].

More recently, toxicological studies about its toxicity have emerged, including genotoxicity, liver degeneration, and an increase in the number of lipid droplets, cytostatic and apoptotic effects, reproductive disturbance by causing sperm impairments, and behavioral changes like hyperactivity in the cubs after in utero exposure [4,9,10,11,12,13,14]. These findings highlight the potential health implications of temephos exposure, particularly in dengue-endemic areas. It was suggested that, in humans, the most probable reaction may be the dephosphorylation of one or both oxon groups of the metabolites, resulting in compounds mentioned above that do not possess acetylcholinesterase (AChE) inhibitory activity [6]. This might be why there are no reports about cholinergic toxicity in humans exposed to this pesticide [15]. Therefore, it was assumed that this larvicide is safe for non-target species.

Temephos has been intensively used during the last five decades in several tropical countries; the information regarding its toxicokinetics and dosimetry is quite limited in humans, making difficult the extrapolation of the toxicological findings observed in animals for human risk assessment. The absence of a biomarker of exposure for its evaluation is a significant gap in our understanding. Therefore, it is urgent to develop a biomarker of exposure to obtain an accurate overview of the exposure in human populations, monitor the effects of this pesticide, and aid in risk assessment. Shafik [8] conducted a study on female rats and administered three consecutive doses of Abate, and urine samples were collected to analyze metabolites. TDP was still detectable in the samples after more than two weeks. However, in this study, the urinary excretion kinetics of temephos and its metabolites were not determined. More recently, it has been proposed that mono-dephosphorylated temephos sulfone (Tem-SO_2_-OH) could be a biomarker of exposure due to its abundance, specificity, and stability in rat blood and tissues [3]. On the other hand, urine is a convenient and affordable sample for most of the population. However, a validated biomarker of exposure to temephos using this matrix has not yet been developed. Additionally, there is no available information on the excretion kinetics of temephos and its metabolites in urine. Also, the samples in that study were processed under extreme pH and temperature conditions, which could have disintegrated other metabolites that may emerge in urine. Thus, the objective of this study was to determine the urinary excretion kinetics of temephos metabolites in adult male Wistar rats following a single oral dose of temephos and propose a biomarker of exposure.

## 2. Materials and Methods

### 2.1. Reagents

Temephos was purified (>97%) from T.M. FOS 500 CE (Agromundo, S.A. de C.V., Mexico City, Mexico) as was described by Verdín-Betancourt et al. [16]. SIDP, Tem-SO, Tem-dox-SO, and Tem-dox-SO_2_ were synthesized and purified from TDP and temephos as previously described by Verdín-Betancourt et al. [3]. TDP, BPS, and β-glucuronidase/sulfatase from *H. pomatia* were obtained from Sigma-Aldrich (St. Louis, MO, USA). HPLC-grade methanol, acetonitrile, water, and dichloromethane were purchased from Fermont (Monterrey, N.L., Mexico). Diethyl ether was obtained from Fisher Scientific (Bridgewater, NJ, USA). Sterile saline solution (NaCl 0.9%) was obtained from PiSA (Guadalajara, Jal., Mexico). Analytical grade granular anhydrous sodium sulfate was purchased from J.T. Baker (Xalostoc, Edo. de México, Mexico). Analytical grade sodium acetate and HPLC grade ethyl acetate were obtained from Merck (Naucalpan, Edo. de México, México). Nitrogen gas was obtained by a Nitrogen Generation System (Agilent Technologies, Santa Clara, CA, USA).

### 2.2. Animals

Adult male Wistar rats (90 days old) were obtained from the Production Unit of Experimental Laboratory Animals (UPEAL, Cinvestav-IPN, México City, México). The animals were housed in filtered acrylic cages and fed ad libitum with Formulab Diet 5008 (PMI Feed Inc., St. Louis, MO, USA) and tap water. The animal room was maintained at 21 ± 1 °C, a relative humidity of 50 ± 10%, and with 12 h light-dark photoperiods. All animal procedures were approved under the ethical principles for ensuring the welfare of experimental animals by the Institutional Committee of Animal Care and Use based on the guidelines of the Mexican Official Norma of Animal Protection (NOM-062-ZOO-1999) based on the Guide for the Care and Use of Laboratory Animals “The Guide”, 2011, NRC, USA. 

### 2.3. Animal Treatment

Animals were individually housed in acrylic metabolism cages for the collection of urine for 8:16 h cycles (inside and outside the cage) for their adaptation during 8 d before treatment. Animals were orally administered with a single dose of temephos (300 mg/kg) emulsified with saline solution (0.9% NaCl). Urine was collected at intervals of 0–8, 8–16, 16–24, 24–48, 60–72, 84–96, 108–120, 132–144, 156–168, 228–240, and 324–336 h. Urine samples were collected and immediately centrifuged at 1600× *g* for 5 min at 4 °C (Eppendorf 5804R, Hamburg, Germany) and stored at −20 °C until processed for analysis.

### 2.4. Extraction of Temephos and Its Metabolites

Temephos urinary metabolites were enzymatically deconjugated, according to Cruz-Hurtado et al. [17], with some modifications. Briefly, 200 µL of urine and 2000 IU of β-glucuronidase/sulfatase were added to a 0.5 M sodium acetate buffer at pH 5 to reach a final volume of 1 mL. The mixture was incubated at 50 °C in a water bath for an hour. To determine the optimal concentration of deconjugated temephos metabolites in the enzymatic hydrolysis assay, the concentration of β-glucuronidase/sulfatase was assessed from 250 to 4000 IU/mL. The enzymatic reaction was stopped by adding 10 mL of methylene chloride and cooling at room temperature; the mixture was vortexed for 1 min and then centrifuged at 1600× *g* for 15 min at room temperature. The organic phase was collected, and another residue extraction was performed using 10 mL of diethyl ether. Both supernatants were pooled and dried using anhydrous sodium sulfate. The solvent evaporated under a nitrogen current. The extracts were stored at −20 °C until analysis by HPLC-DAD. Extracts were dissolved in 200 µL of acetonitrile immediately before analysis. Urinary levels of temephos metabolites were adjusted by creatinine, which was determined using a commercial kit from Randox (CR510, Randox Laboratories Ltd., Crumlin, UK).

### 2.5. Analysis of Temephos and Its Metabolites by Liquid Chromatography

Temephos and its metabolites were analyzed by injecting urinary extracts into a liquid chromatograph equipped with a quaternary pump, autosampler, degasser, and UV-Vis Diode-Array detector (Agilent 1200, Agilent Technologies, Palo Alto, CA, USA). A ZORBAX Eclipse XDB C-18 column (4.6 × 150 mm, 5 µm) was used (Agilent Technologies, Deerfield, IL, USA). The detector wavelength was set at 254 nm, the reference wavelength was 550 nm, and the mobile phase was modified from Verdín-Betancourt et al. [16]; it consisted of methanol (A), acetonitrile (B), and water (C), with initial solvent conditions of 27% A, 8% B, and 65% C at a rate of 1 mL/min at 25 °C. After injecting the sample (with different volumes), a 2.5 min linear gradient change was applied to achieve 23.5% A, 12.5% B, and 64% C, which was maintained for 1 min, followed by a second 2.5 min linear gradient change to 18% A, 18% B, and 64% C. Afterwards, another 4 min linear gradient change was applied to reach 20% A, 30% B, and 50% C. This condition was maintained for 2 min. Then, a fourth gradient change was applied, achieving 70% A, 25% B, and 5% C. In the end, the initial conditions were re-established to 27% A, 8% B, and 65% C. The column was equilibrated for 2 min before the next injection. The identity of temephos and its metabolites were confirmed by their retention time (*t*_ret_) and UV spectra of standards. The concentrations of temephos, TDP, TDP-SO, and BPS were determined using calibration graphs of each compound. The concentration of the non-identified metabolite (NIM) was estimated using the Tem-dox-SO calibration graph due to their similarity in the UV spectrum and *t*_ret_ [16].

### 2.6. Recovery of Temephos Metabolites

The recovery of metabolites (TDP, SIDP, and BPS) was evaluated using rat urine spiked with 2, 10, and 50 µg/mL of standards. The samples were processed and analyzed as described before. Recovery was obtained by the percent of the compound mass detected in the spiked samples. The precision of the assays was evaluated by calculating the relative standard deviation (coefficient of variation). Assays were carried out in three urine samples from non-treated rats, which were analyzed by duplicate. The limit of detection (LOD) and the limit of quantification (LOQ) were determined using the following equations: LOD = 3.3 × SD/*m* and LOQ = 10 × SD/*m*. The SD (standard deviation) for each metabolite was calculated by injecting seven replicates of the lowest concentration of standard of the calibration graph, and *m* is the slope of the calibration graph [18].

### 2.7. Toxicokinetic Parameters of Temephos Metabolites

The urinary elimination constant (*K*_Elim-U_) to determine the half-life of elimination (*t*_1/2 Elim-U_) for TDP, BPS, and NIMs was estimated from the slope of the curves in the Rate of Excretion plots (log-linear Rate of excretion (ΔU/Δt) versus mid-point time). Other toxicokinetic parameters such as the area under the curve (AUC), maximal concentration (*C*_max_), and maximal time (*t*_max_) were calculated after fitting the mean concentration data to a non-compartmental model using PK Solution 2.0 software (Summit Research Services, Montrose, CO, USA). The normality test of data was carried out using Shapiro–Wilk’s test. Statistical differences in the different experimental conditions were assessed by Student’s “*t*” test. Statistical analyses and plots were performed using SigmaPlot Ver. 13.0 (Systat Software, Inc., Richmond, CA, USA).

## 3. Results

Our research, based on the work of Verdín-Betancourt et al. [16], carefully adjusted the mobile phase to enhance the detection of other polar analytes in urine extracts from rats treated with temephos. While improving peak resolution and detection, these adjustments also caused a delay in *t*_ret_ elution compared to previous reports. The order of elution remained unaffected by these modifications. The quantification of peaks was carried out by the external standard method, which included six-point calibration graphics in a range of 10–500 ng. Linear relationships (*r*^2^ > 0.9976) were observed for all analytes. Equations obtained by the linear regression of analyte mass versus peak area were as follows: TDP, *y* = 3.31*x* − 4.9; SIDP, *y* = 1.34*x* − 1.34; BPS, *y* = 3.65*x* − 2.22. The detection limits for TDP, SIDP, and BPS were 0.020, 0.200, and 0.025 µg/mL, respectively. The limits of quantification were 0.055, 0.600, and 0.080 µg/mL, respectively. Recoveries for BPS and TDP ranged from 74.3 to 100.6% in the urine matrix, and the precision of the method was less than 11.7% (Table 1).

**Figure 2:**
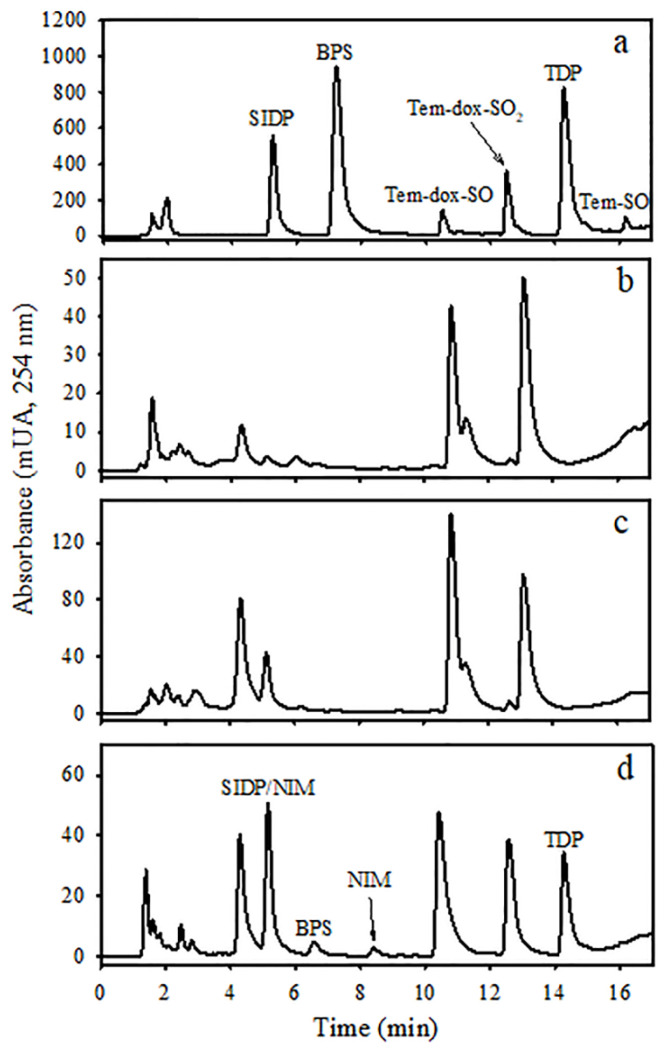
HPLC-DAD chromatograms of (**a**) standard solutions of temephos metabolites (10 µg/mL); (**b**) urine extract of non-treated rats without β-glucuronidase/sulfatase enzymatic hydrolysis; (**c**) urine extract of non-treated rat with β-glucuronidase/sulfatase enzymatic hydrolysis; and (**d**) urine extract of temephos-treated rat with β-glucuronidase/sulfatase enzymatic hydrolysis. Urine samples were processed according to the procedures described in Section 2. The organic phase was dried under a stream of N_2_, and the residue was dissolved in 200 µL of acetonitrile.

**Figure 3:**
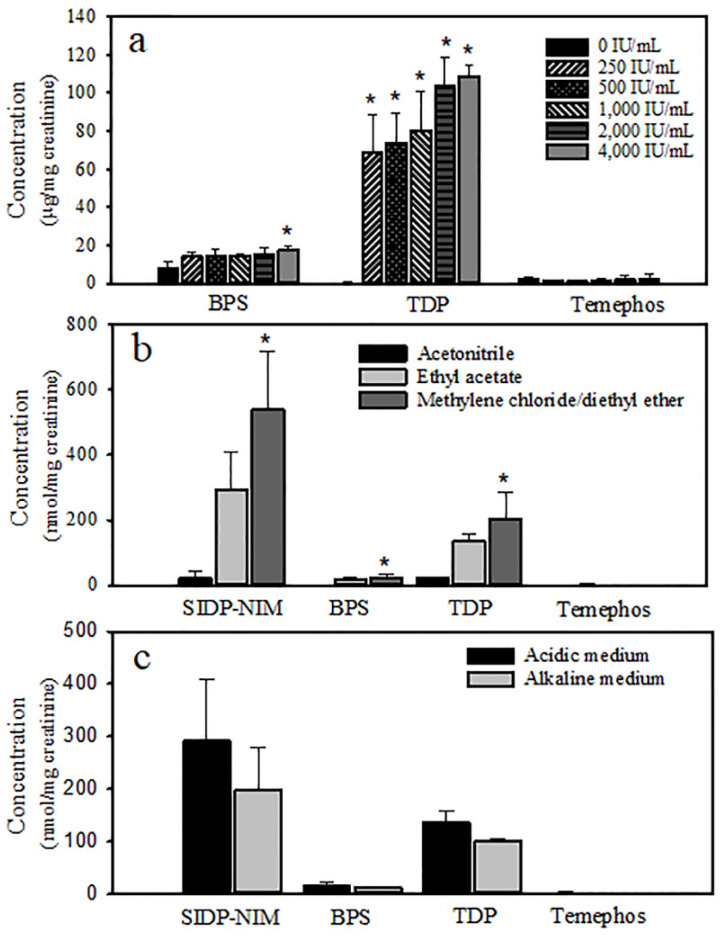
Experimental conditions for extracting temephos metabolites in the urine of temephos-treated rats: (**a**) β-glucuronidase/sulfatase concentration; (**b**) liquid–liquid extraction using acetonitrile, ethyl acetate, or methylene chloride/diethyl ether; and (**c**) extraction in acidic or alkaline medium. Urine samples were processed according to the procedures described in Section 2. The organic phase was dried under a stream of N_2_, and the residue was dissolved in 200 µL of acetonitrile and analyzed by HPLC-DAD. The values shown represent the mean ± SD. * *p* < 0.05, Student’s “*t*” test (n = 3).

SIDP, BPS, and TDP metabolites eluted at 5.2, 7.2, and 14.2 min, respectively, as well as temephos eluted at 17.2 min (Figure 2a). Additional peaks associated with internal metabolites unrelated to temephos exposure appeared at 4.4, 5.1, 10.9, and 13.1 min (Figure 2b). Chromatograms of urine extracts of non-enzymatically hydrolyzed metabolites showed some unknown peaks, and only BPS was easily quantifiable (Figure 2c). After enzyme hydrolysis using β-glucuronidase/sulfatase in the enzyme assay ranging from 200 to 400 IU/mL incubated at 50 °C for 1 h, levels of SIDP, BPS, and TDP markedly increased in urine extracts (Figure 3a). TDP formation increased more than 60-fold at all enzyme concentrations, and its maximum was reached at 2000 IU/mL. BPS increased about 2-fold but was only significant at 4000 IU/mL. The peak of SIDP (*t*_ret_ 5.2 min) corresponds to the co-elution of SIDP and an internal metabolite, which also increases after enzymatic hydrolysis. Therefore, it was challenging to quantify this metabolite despite efforts to separate them by modifying the polarity of solvents and chromatographical conditions. Temephos metabolites were better extracted firstly using methylene chloride followed by diethyl ether compared with acetonitrile or ethyl acetate (Figure 3b). Likewise, in acidic conditions, metabolites were better extracted than in the alkaline medium (Figure 3c). Temephos was practically undetectable in urine samples with and without enzyme hydrolysis and the solvent used for the extraction.

**Figure 4:**
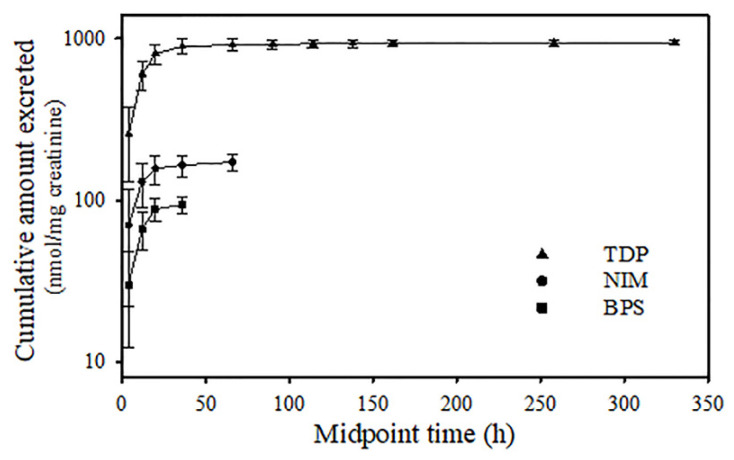
Cumulative amount of temephos metabolites excreted in rat urine after an oral dose administration of temephos (300 mg/kg). The data points represent the cumulated amount of the metabolite excreted during specific time intervals. The excretion for each metabolite was calculated based on the urine total volume and concentration collected at different time intervals after dosing. The values shown represent the mean ± SD (n = 7).

**Figure 5:**
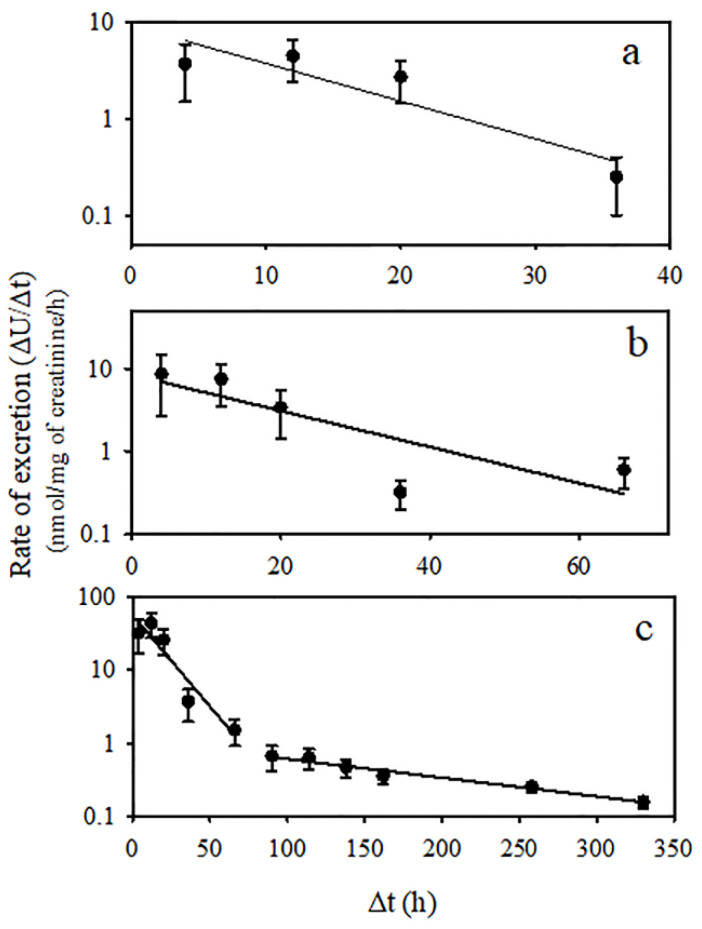
Semi-log plot of ΔU/Δt vs. midpoint time of temephos metabolites excreted in urine following administration of a single oral dose of temephos (300 mg/kg). The metabolites measured were as follows: (**a**) BPS; (**b**) NIM; and (**c**) TDP. The excretion rate was calculated by dividing the total amount excreted during each interval by the length of the time interval between sample collections. Each point corresponds to the midpoint of the time interval. The values shown represent the mean ± SD (n = 7).

The average urinary concentrations of temephos metabolites were systematically measured at different time intervals following administration, and these data were utilized to construct a plot of cumulative excretion (Figure 4). TDP emerged as the primary metabolite and may be detected up to 320 h after dosing. Additionally, NIMs and BPS were detected for 70 and 40 h, respectively. Urinary elimination kinetics of TDP, BPS, and the NIM are shown in Figure 5. BPS and the NIM kinetics showed a monophasic decline; however, TDP exhibited a two-compartment model (Figure 5c). Toxicokinetic parameters of temephos metabolites are shown in Table 2. The *C*_max_ of TDP was 366 nmol/mg of creatinine, occurring at 9 h after temephos administration. This concentration was 4.2- and 8.9-fold higher than those observed for NIMs and BPS, which reached their *t*_max-U_ at 7 and 9 h after dosing, respectively. The AUC_0–∞_ for TDP was 297,540 ± 76,013 nmol-h/mg of creatinine, values much higher than those of NIMs and BPS. The *t*_1/2 Elim-U_ of NIMs and BPS were 31.7 and 17.7 h, respectively. In the case of TDP, the *t*_1/2 Elim-U_ in the first phase was 27.8 h, and, for the 2nd phase, was 272.1 h.

## 4. Discussion

Temephos is the main larvicide used for approximately five decades to combat dengue, Zika, and chikungunya vectors. Since the 1970s, the WHO has recommended its use in different bodies of water, including those intended for human consumption, at a concentration that does not exceed 1 mg/L. This concentration does not change the taste of the water and has been considered to have low acute oral toxicity [1]. However, recent studies indicate that temephos can produce reproductive damage [4,13] and induce abnormal behavior and social interactions after prenatal exposure [14], and its oxidized derivatives inhibit AChE activity [16]. All these antecedents indicate that exposure to temephos represents a risk for populations. In Mexico and other tropical countries, it is the first pesticide of choice in abatement campaigns in dengue-endemic areas [19]. Despite its wide use, no information is available about exposure in humans due to the lack of a biomarker of exposure associated with an effect in exposed populations.

The current study depicts the kinetics of urinary excretion of temephos metabolites TDP, BPS, and NIMs. TDP was the major metabolite of temephos and was excreted mainly in conjugated form, followed by the NIM and BPS, which are excreted in free and conjugated forms. Although the SIDP metabolite was also detected, it cannot be precisely determined due to the overlap in the chromatograms of the corresponding peak with a physiological metabolite from the rat. These findings agree with those reported by Blinn [7] and Shafik [8], who observed that TDP is the major metabolite followed by SIDP and BPS in rat urine after the administration of either one or three doses of this larvicide. Preliminary results suggest that the NIM could correspond to temephos sulfoxide monohydrolized. The definitive identification of this metabolite and the determination of SIPD can be made using another analytical tool, such as mass spectrometry, which is the subject of further investigation.

The results of this study confirm that temephos is efficiently metabolized and excreted in the urine. TDP, SIDP, BPS, and the NIM were detected in the urine during the first 8 h after dosing. Their levels gradually decreased until they were undetectable, and only TDP was detected after 72 h. The information on the urinary metabolite toxicokinetics is very limited. Shafik [8] reported the excretion of this metabolite more than two weeks after the administration of temephos for three consecutive days. Likewise, Blinn [7] reported that TDP was the principal metabolite in urine. In the present study, TDP was excreted in urine, clearly exhibiting a two-compartment model, each one with a different value of slope in the rats administered with a single dose of 300 mg/kg of temephos (Figure 5). The *t*_1/2 Elim-U_ was estimated to be 27.8 h in the first phase and 272.1 h in the second largest phase. A two-compartment model with time lag was proposed to describe the pharmacokinetics of xenobiotics subject to enterohepatic circulation [20]. In this regard, Blinn [7] suggested that the biliary metabolic route for temephos may be a significant factor in the total guinea pig metabolic scheme based on the high levels of the pesticide in the bile compared with blood levels, which suggest an enterohepatic circulation. This proposal may apply to TDP, considering that temephos is stored in adipose tissue and gradually released into the blood to be metabolized and eliminated in the urine, as previously reported [3]. BPS and the NIM were analyzed using a one-compartment model. Their *t*_1/2 Elim-U_ were 17.7 and 31.7 h, and their detection was difficult after 48 and 72 h, respectively. Altogether, the data help explain how TDP can be easily detected for many days post-dosing. However, the oxidation of the central sulfur atom to form sulfinyl- and sulfonyl-derivatives is not discarded, although they could be at trace levels because its determination was difficult.

The formation of TDP indicates the biotransformation of temephos in a series of consecutive reactions. The canonical pathway needs oxon formation with consecutive hydrolysis. Nevertheless, the TDP formation could occur directly (without oxon formation), releasing dimethyl dithiophosphate (DMDTP), as has been observed in simultaneous exposures to various pesticides, including temephos [21]. On the other hand, if temephos initially suffers one or two oxidation reactions in the central sulfur, Tem-SO and Tem-SO_2_ are formed, which subsequently will continue to the formation of oxons and the hydrolysis of the phosphodiester to generate phenolic derivatives of SIDP and BPS, respectively. Finally, they are conjugated as sulfates or glucuronides to be secreted into the bloodstream or the bile and excreted by urine or feces (Figure 1). During temephos biotransformation, certain intermediary metabolites can be conjugated with sulfate or glucuronide. Blinn [7] found that phenolic metabolites of temephos are primarily excreted as sulfate esters. To identify most of these byproducts, we carried out the enzymatic hydrolysis using β-glucuronidase/sulfatase under controlled conditions, enabling us to detect the NIM, in addition to TDP, SIDP, and BPS. All possibilities indicate that temephos has several options for metabolization, generating several intermediary metabolites and forming TDP as the preferential way to eliminate temephos.

The proposed metabolic pathway of temephos may be involved in mixed-function microsomal monooxygenases (MFOs), particularly cytochrome P450s (CYPs) and flavin-containing monooxygenases (FMOs). Although CYPs would be expected to catalyze temephos biotransformation, the information demonstrating this occurrence is scarce, as has been demonstrated for other organophosphorothioate pesticides (OPTs) [22,23]. CYPs and FMOs could also be involved in the *S*-oxidation of the sulfur central atom to produce sulfoxides and sulfones, which have been considered as the formation of less toxic metabolites from OPs [24,25]. In the same sense, another reaction of the detoxification of temephos could be the oxon hydrolysis, which leads to its dearylation to produce alkyl phosphate, plus phenolic products catalyzed by plasma and hepatic esterases and hepatic CYPs, which could even directly carry out the dearylation without the formation of oxons [26,27,28]. It is essential to consider that internal and external factors may produce variations in enzyme activities involved in the temephos biotransformation and consequently may affect the rate and profile of metabolites. In this sense, Ferguson et al. [29] reported that temephos inhibits FMOs but does not induce hepatic CYP expression, which suggests a possible inhibition of CYP isoforms acting as a suicide substrate, such as has been demonstrated for other OPTs [30,31]. Likewise, Murphy and Sheever [32] reported the inhibition of carboxylesterases by temephos. Therefore, repeated exposure to temephos may affect its own biotransformation and provoke metabolic interactions with other xenobiotics. Further studies are needed to characterize enzymes involved in temephos biotransformation and how they are affected by this pesticide and produce metabolic interactions.

TDP is the major metabolite of temephos; it is excreted nearly as a conjugated metabolite in the urine and can be easily detected in this biological sample after enzymatic hydrolysis using β-glucuronidase/sulfatase. In addition, it is a specific metabolite of temephos. Although, it is important to consider that it is an environmental pollutant and a surrogate of bisphenol A (BPA) [33,34]. It can be easily determined two weeks after exposure based on its very large *t*_1/2 Elim-U_ of the second elimination phase. Based on these criteria, TDP may be used as a biomarker of exposure temephos, including humans. The extrapolation of the biotransformation pathway proposed from the rat to the human appears limited due to the differences in the enzymes present in each species. However, similar metabolic products may also be found despite different CYP isoforms expressed in different species [35]. This proposal is supported by results from an in silico study on temephos biotransformation, assuming a similar pathway in humans [6]. Therefore, more studies are needed to determine the definitive biotransformation pathway in humans and to assume a similar toxicokinetic.

TDP, BPS, and SIDP are the main metabolites of temephos in urine and blood after administering only one dose or repeated doses [3,4,7,8]. TDP and BPS are BPA analogs; they are used as substitutes for BPA in several applications and promote endocrine and immune disruption based on their properties to interact with the estrogen receptor (ER), in addition to causing oxidative stress by releasing reactive oxygen species (ROS) [33,34,36]. However, based on its chemical structure, SIDP could also be included in this classification, in addition to being another essential metabolite of temephos. These findings all indicate that temephos exposure represents a potential source of bisphenol compounds contributing to human exposure to endocrine and immune disruptors.

In summary, temephos is extensively metabolized to phenolic products, eliminated as non-conjugated or/and sulfate and glucuronide conjugates in the urine. TDP is the principal temephos metabolite; it is specific and stable. Its urinary toxicokinetic property was fitted to a two-compartmental model, and its long half-lives allows its long-term detection after exposure. Assuming some toxicokinetics and metabolic similarities between rats and humans, TDP is an excellent candidate for use as a biomarker of temephos exposure. However, more studies are needed to determine the metabolic pathway of temephos in humans. The formation of oxons and phenolic products from temephos biotransformation may contribute to a better understanding of the adverse effects associated with temephos exposure. It is important to note some limitations of this study. Under the experimental conditions used, we could not fully characterize the urinary toxicokinetics of SIDP because it overlapped with a rat endogenous metabolite, and the NIM was not chemically identified. Further studies are needed using different experimental conditions and more sensitive and specific analytical systems, such as mass spectrometry. This will help improve the application of findings in future studies.

## 5. Conclusions

The experimental conditions for analyzing temephos metabolites in rat urine were established using HPLC-DAD. The metabolites found in rat urine are TDP, SIDP, BPS, and NIMs. TDP is excreted mostly in its conjugated form, so enzymatic hydrolysis with β-glucuronidase/sulfatase is necessary for its determination. TDP-SO and BPS are excreted in both free and conjugated forms. The urinary excretion kinetics of TDP showed biphasic behavior, while those of BPS and NIMs behaved in a monophasic manner. TDP is a good candidate for use as a biomarker of temephos exposure.

## Figures and Tables

**Table 1:** Recovery and precision of temephos metabolites in spiked urine at different concentrations.

	Concentration (µg/mL)					
Metabolite	2		10		50	
	Recovery (%)	Precision (%)	Recovery (%)	Precision (%)	Recovery (%)	Precision (%)
BPS	74.3 ± 12.9	3.3	100.6 ± 12.9	5.2	92.8 ± 10.3	4.9
TDF	90.5 ± 15.9	11.7	100.3 ± 19.8	3.5	87.4 ± 12.6	7.0

Note: Aliquots of urine (200 µL) were spiked with standards and processed for HPLC analysis as described in Section 2. Recovery represents (mean ± SD) % of the mass of chemical detected in the spike samples. Precision represents the coefficient of variation in the assay. Values shown represent the mean ± SD (n = 3).

**Table 2:** Urinary toxicokinetic parameters of temephos metabolites of male adult rats treated with an oral administration of temephos.

Metabolite	*K*_Elim-U_ (h^−1^)		*t*_1/2 Elim-U_ (h)		AUC_0–∞_ (nmol-h/mg Creatinine)	*C*_max-U_ (nmol/mg Creatinine)	*t*_max-U_ (h)
	1st Phase	2nd Phase	1st Phase	2nd Phase			
BPS	-	0.03912	-	17.7	2455 ± 974	41 ± 16	9 ± 6
NIM	-	0.02188	-	31.7	9541 ± 3843	88 ± 40	7 ± 4
TDP	0.02496	0.00255	27.8	272.1	297,540 ± 76,013	366 ± 115	10 ± 4

## Data Availability

The original contributions presented in this study are included in the article. Further inquiries can be directed to the corresponding author(s).
